# Patient-Reported Burden of Illness in a Prevalent COPD Population Treated with Long-Acting Muscarinic Antagonist Monotherapy: A Claims-Linked Patient Survey Study

**DOI:** 10.1007/s41030-019-0091-0

**Published:** 2019-04-01

**Authors:** Beth Hahn, Richard H. Stanford, Alyssa Goolsby Hunter, Breanna Essoi, John White, Riju Ray

**Affiliations:** 1 US Value Evidence and Outcomes, GSK, 5 Moore Drive, Research Triangle Park, NC 27709-3398 USA; 2 Health Economics and Outcomes Research, Optum, 11000 Optum Circle, Eden Prairie, MN 55344 USA; 3 US Medical Affairs, GSK, 5 Moore Drive, Research Triangle Park, NC 27709-3398 USA

**Keywords:** Bronchodilator agents, COPD, Health surveys, Patient-reported outcomes

## Abstract

**Introduction:**

Symptom burden in inadequately controlled chronic obstructive pulmonary disease (COPD) considerably impacts quality of life, healthcare resource utilization (HCRU) and associated costs. This claims-linked cross-sectional survey study assessed symptom burden and HCRU among a prevalent population of COPD patients prescribed long-acting muscarinic antagonist (LAMA) monotherapy.

**Methods:**

Patients were identified using claims data from the Optum Research Database. Eligible patients were aged ≥ 40 years with 12 months’ continuous enrollment in a US health plan, ≥ 2 medical claims containing COPD diagnosis codes ≥ 30 days apart, and ≥ 2 claims for LAMA monotherapy in the latter half of the 12-month sample identification period. Patients were mailed a cross-sectional survey assessing patient-reported outcomes (PROs) [COPD assessment test (CAT) and modified medical research council dyspnea scale (mMRC)], clinical characteristics, smoking history, and demographics. Patients also completed the Exacerbations of Chronic Pulmonary Disease Tool (EXACT-PRO) daily diary for 7 days. HCRU was assessed from claims data.

**Results:**

The study included 433 patients with a self-reported healthcare provider COPD diagnosis, and both claims-based and self-reported LAMA monotherapy treatment (mean age 71.0 years; 59.8% female). Most patients (85.5%) reported a high symptom burden (CAT score ≥ 10), 45.5% had high levels of dyspnea (mMRC grade ≥ 2), and 64.4% reported more severe daily symptoms by the EXACT-PRO. Most patients (71.6%) reported high scores on ≥ 2 PROs. More patients with high symptom burden had COPD-related emergency department visits than those with lower disease burden (27.6% vs 12.7%, *P *= 0.012).

**Conclusions:**

In conclusion, a large proportion of patients with COPD receiving LAMA monotherapy experienced a high symptom burden and may benefit from therapy escalation. Healthcare professionals can use validated PROs to help them assess symptom burden.

**Funding:**

GlaxoSmithKline (GSK study number: 205862)

**Electronic supplementary material:**

The online version of this article (10.1007/s41030-019-0091-0) contains supplementary material, which is available to authorized users.

## Introduction

Chronic obstructive pulmonary disease (COPD) is one of the most common chronic diseases, and is a leading cause of mortality and morbidity worldwide [1, 2]. In the USA, it is the third leading cause of death and is reported to affect over 15 million people [3, 4]. This number is expected to rise due to increasing exposure to risk factors and changing population demographics. COPD, characterized by airflow obstruction that progressively worsens over time, leads to debilitating symptoms such as dyspnea and persistent cough and is one of the leading causes of hospitalizations and emergency department (ED) visits globally [1, 5]. COPD is also associated with considerable economic burden, with COPD exacerbations in particular contributing significantly to both direct and indirect healthcare costs [1, 2, 5].

The mainstay of pharmacological therapy for COPD is bronchodilation with a long-acting muscarinic antagonist (LAMA), a long-acting β_2_-agonist (LABA), or a combination of the two [6–8]. Currently, the 2019 Global Initiative for Chronic Obstructive Lung Disease (GOLD) strategy document recommends LAMA or LABA monotherapy as the initial therapy for patients with COPD with a lower symptom burden and higher exacerbation history, and a higher symptom burden and lower exacerbation history [2]. However, a significant proportion of patients can fail to achieve adequate control of symptoms when treated with LAMA or LABA monotherapy [9]. For these patients, escalation to LAMA/LABA combination therapy is recommended, or escalation to triple therapy [a combination of a LAMA, LABA, and inhaled corticosteroid (ICS)] [3] for patients at higher risk of exacerbation [2, 9].

The increased symptom burden in patients with inadequately controlled COPD can reduce activity levels and quality of life (QoL) [10–12], as well as increasing the risk and frequency of exacerbations which are associated with more rapid disease progression [13] and are a major driver of healthcare resource utilization (HCRU) and associated costs [14, 15]. It is therefore important to understand the symptom burden for patients receiving COPD treatment, so that treatment strategies can be optimized.

The objective of this study was to further understand the burden of COPD by examining symptom burden and HCRU among a prevalent population of patients with COPD treated with LAMA monotherapy. The primary objective was to identify the proportion of patients reporting COPD symptoms while receiving treatment with LAMA monotherapy. Secondary objectives included the description of the patient-reported burden of illness, and all-cause and COPD-related HCRU.

## Methods

### Study Design

The study was a claims-linked, cross-sectional survey of patients with COPD who were prescribed LAMA monotherapy and enrolled in commercial or Medicare Advantage (MA) insurance plans. Patients were identified using medical and pharmacy claims, and enrollment data from the Optum Research Database (ORD) between October 1, 2015 and September 30, 2016. The ORD is a large, geographically diverse, US administrative claims database. In 2016, approximately 32.8 million individuals with commercial coverage and 3.2 million individuals with MA coverage were included in the ORD.

Patients who met study inclusion/exclusion criteria (below) were recruited directly by mail and consented to study participation by returning a completed paper survey and/or a 7-day daily diary. Survey data collection occurred from October to December 2016 and was conducted using a modified Dillman method [16]. Patients were paid $25 following the return of the survey and/or diary with a maximum payment of $50 per patient.

The study was approved by the New England Institutional Review Board (NEIRB), on September 9, 2016 (IRB #120160900). Data collection activities were initiated following all approvals. All procedures performed in studies involving human participants were in accordance with the ethical standards of the institutional and/or national research committee and with the 1964 Helsinki declaration and its later amendments or comparable ethical standards. Informed consent to take part in the study was implied by the return of study materials.

### Patient Identification

Patients were required to be at least 40 years of age and continuously enrolled in a commercial or MA health plan with both medical and pharmacy benefits during the 12-month baseline period. Patients were also required to have ≥ 2 medical claims containing diagnosis codes commonly used to define COPD [2, 17] [International Classification of Disease 10th Revision Clinical Modification (ICD-10-CM) codes J40-J44] at least 30 days apart during the 12-month baseline and ≥ 2 claims for LAMA monotherapy (umeclidinium, tiotropium, or aclidinium) in the latter 6 months of the sample identification period (codes and treatments are presented in Supplementary Table S1). Patients were excluded if they had prescription claims for any ICS- or LABA-containing therapy (ICS, ICS/LABA, or LAMA/LABA) during the 12-month baseline period. Patients with evidence of lung cancer during the baseline period were excluded. All patients were also required to self-report a healthcare professional diagnosis of COPD and LAMA monotherapy use, and to be able to complete the study surveys in English.

### Study Measures

Demographic, sociodemographic, and clinical characteristics were captured using both patient-reported survey data and claims data, including patient-reported time since diagnosis, and smoking status. Claims-based evidence of 11 COPD-related comorbidities were identified: dyspnea; hypertension; atherosclerotic cardiovascular disease (ASCVD); type 2 diabetes mellitus; obstructive sleep apnea; depression; anxiety (all comorbidities were based on diagnosis codes except for depression and anxiety rates, which were based on evidence of diagnosis and/or treatment) [18]. Quan–Charlson comorbidity scores [19] were calculated based on the presence of diagnosis codes on medical claims during the baseline period. HCRU, including ambulatory (physician office and outpatient), inpatient, and ED visits, were obtained from medical claims. HCRU was defined as COPD-related if the medical claim included an ICD-10-CM diagnosis code for COPD in any position.

### PRO Measures

In this study, burden of illness for COPD was defined by symptom burden, dyspnea, and symptom severity, and was assessed using three COPD-related validated patient-reported outcomes (PRO) measures. Symptom burden was assessed using the COPD Assessment Test (CAT) [20] and dyspnea was assessed using the modified medical research council dyspnea scale (mMRC) (both assessed using survey data); symptom severity was measured using the EXACT [21] (assessed using a daily diary for 7 days). The EXACT was used in a time-limited fashion to assess symptoms not addressed by CAT or mMRC, and was not used to evaluate exacerbation history. Classifications of burden of illness, dyspnea, and symptom severity were based on established cut-points, where available: [2] patients with an mMRC score ≥ 2 (range 0–4) were classified as having severe dyspnea, and patients with a CAT total score ≥ 10 (range 0–40) were classified as having a high symptom burden [22]. For EXACT scores, patients with more severe symptoms were defined as those having an EXACT score greater than the mean total score in the study sample on at least 1 day. The proportion of patients with CAT, mMRC, and EXACT scores meeting these thresholds were used to show the prevalence of high symptom burden among patients treated with LAMA monotherapy.

### Statistical Analyses

The analytic population consisted of respondents with claims-linked survey and diary data who met all study inclusion and exclusion criteria (*n* = 433). Statistical analyses were performed using SAS software (SAS Institute Inc., Cary, NC, USA, version 9.4) on a Unix platform. Results are presented descriptively. Statistical comparisons were performed using the appropriate two-sided tests (e.g., *t*-test, chi-square test) based on the distribution of the measure. For all PRO measures, the mean total, summary, and/or domain scores and standard deviations (SDs) were calculated. In all analyses, statistical significance was defined as *P *< 0.05.

## Results

### Study Population

**Fig. 1:**
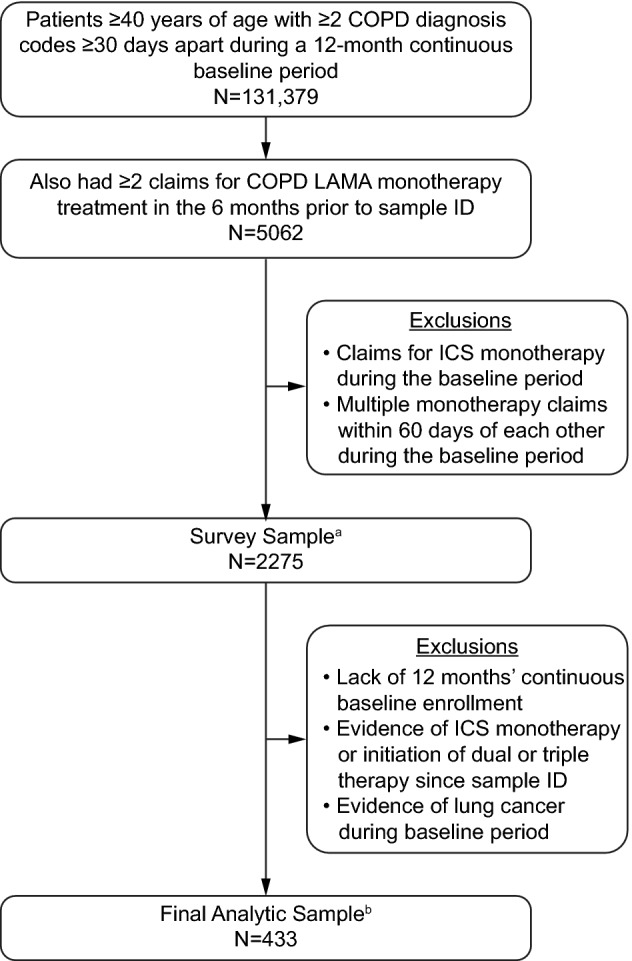
Patient disposition. ^a^Patients who were randomly chosen and invited to participate in the survey study by mail; ^b^patients with matched claims, survey, and diary data. *COPD* chronic obstructive pulmonary disease; *ICS* inhaled corticosteroid; *ID* identification; *LAMA* long-acting muscarinic antagonist

A total of 2275 patients met the eligibility criteria for the study [including having multiple medical claims containing COPD diagnosis codes (Supplementary Table S1), a self-reported healthcare provider COPD diagnosis, and both claims-based and self-reported LAMA monotherapy treatment] and were invited to participate. Of these, 528 completed the survey and daily diary (29.8% response rate [23]) and 433/528 had matched claims, survey, and diary data, and were included in the final analyses (Fig. 1).

Patient demographics and clinical characteristics are presented in Table 1. Over half of the patients were female (59.8%), and the average age was 71.0 years. The majority had an education level of high school or less (58.4%), annual household income < $50,000 (79.2%), and were current or former smokers (92.6%). There was a high incidence of comorbidities during the 12-month baseline period: all patients had at least one comorbidity, as measured by the Quan–Charlson comorbidity index, [19] with a mean (SD) baseline score of 2.2 (1.6), and with 36% of patients having a mean comorbidity score of ≥ 3. A comparison of baseline demographics of respondents and non-respondents to the survey is shown in Supplementary Table S2. Respondents and non-respondents tended to be similar when compared by select demographic characteristics (age, gender, geographic region).

**Table 1:** Demographic, sociodemographic, and clinical characteristics

	Total (*n *= 433)
Age^a^, mean (SD)	71.0 (9.4)
Female^a^, *n* (%)	259 (59.8)
Insurance type^b^, *n* (%)	
Commercial	51 (11.8)
Medicare advantage	382 (88.2)
Race^a,c^, *n* (%)	
Black or African American	31 (7.2)
White	375 (86.8)
Education level^a^, *n* (%)	
High school or less	247 (58.4)
College/graduate school	176 (41.6)
Missing	10
Household income^a^, *n* (%)	
< $50,000	339 (79.2)
≥ $50,000	48 (11.2)
Declined to answer	41 (9.6)
Missing	5
Time since COPD diagnosis^a^, *n* (%)	
≤ 5 years	226 (52.4)
> 5 years	205 (47.6)
Missing	2
Quan–Charlson comorbidity score^b^, mean (SD)	2.2 (1.6)
COPD-related comorbidities^b,d^, *n* (%)	
Hypertension	330 (76.2)
Dyspnea	166 (38.3)
Depression (diagnosis and/or treatment)	154 (35.6)
Anxiety (diagnosis and/or treatment)	145 (33.5)
ASCVD	133 (30.7)
T2DM	115 (26.6)
Smoking status^a^, *n* (%)	
Current smoker	122 (28.2)
Former smoker	279 (64.4)
Never smoked/lives with smoker	15 (3.5)
Never smoked/no household smoke	17 (3.9)
Missing	0
Smoking pack years^a,e^, mean (SD), *n*	46.9 (31.2), 381
Geographic region^b^, *n* (%)	
Northeast	77 (17.8)
Midwest	125 (28.9)
South	188 (43.4)
West	43 (9.9)

Percentages were calculated based on respondents with available data

*ASCVD* atherosclerotic cardiovascular disease, *COPD* chronic obstructive pulmonary disease, *SD* standard deviation, *T2DM* type 2 diabetes mellitus

^a^Survey-based; ^b^claim-based; ^c^0.9% to 2.1% reported identification as American Native, Asian, Multi-racial, or other race; ^d^comorbidities with incidence < 20% not shown; ^e^number of cigarettes smoked per day*number of years of smoking/20

### COPD Burden of Illness

Despite all patients receiving LAMA monotherapy, the majority reported a high COPD symptom burden; 85.5% and 39.0% had a CAT total score ≥ 10 or ≥ 21, respectively, and the mean (SD) overall CAT score was 18.5 (8.4). In addition, almost half of the patients (45.5%) had high levels of dyspnea (mMRC grade 2–4); the mean (SD) mMRC score was 1.6 (1.0), and most patients experienced shortness of breath with less than strenuous exercise (90.5%). Analysis of the EXACT daily diary scores showed that 64.4% of patients had more severe symptoms (relative to the mean total study sample score on at least 1 day) and the mean (SD) average EXACT total score was 37.1 (12.1) (Table 2).

**Table 2:** COPD-related patient-reported outcomes

	Total (*n *= 433)
CAT total score, mean (SD)	18.5 (8.4)
CAT^a^, *n* (%)	
CAT total score < 10	63 (14.6)
CAT total score ≥ 10	370 (85.5)
CAT total score 10–20	201 (46.4)
CAT total score 21–30	127 (29.3)
CAT total score 31–40	42 (9.7)
mMRC score, mean (SD)	1.6 (1.0)
mMRC categories^b^, *n* (%)	
Low dyspnea severity (grades 0–1)	236 (54.5)
High dyspnea severity (grades 2–4)	197 (45.5)
EXACT total score, mean (SD)	37.1 (12.1)
EXACT categories^c^, *n* (%)	
Less severe symptoms	154 (35.6)
More severe symptoms	279 (64.4)
PRO combinations, *n* (%)	
Low CAT score/low mMRC score/low EXACT score	52 (12.0)
High CAT score/low mMRC score/low EXACT score	61 (14.1)
Low CAT score/high mMRC score/low EXACT score	3 (0.7)
Low CAT score/low mMRC score/high EXACT score	7 (1.6)
High CAT score/high mMRC score/low EXACT score	38 (8.8)
High CAT score/low mMRC score/high EXACT score	116 (26.8)
Low CAT score/high mMRC score/high EXACT score	1 (0.2)
High CAT score/high mMRC score/high EXACT score	155 (35.8)

*CAT* COPD Assessment Test, *COPD* chronic obstructive pulmonary disease, *EXACT* Exacerbations of Chronic Pulmonary Disease Tool, *mMRC* modified Medical Research Council dyspnea scale, *PRO* patient-reported outcome, *SD* standard deviation

^a^Higher CAT values indicate greater symptom burden; ^b^higher mMRC values indicate greater dyspnea; ^c^EXACT symptom categories: severe symptoms = having an EXACT score greater than the mean total score in the study sample, on at least 1 day; higher EXACT values indicate greater symptom severity

Over one-third (35.8%) of patients had a higher symptom burden on all three PRO measures (mMRC, CAT, and EXACT), and 71.6% reported high scores on ≥ 2 COPD-related PRO measures. By contrast, only 12.0% of patients experienced low symptom burden on all three measures (Table 2).

### All-Cause HCRU

Patients were prescribed a mean (SD) of 12.8 (6.7) unique medications during the 12-month baseline period. Patients with more severe disease burden (CAT score ≥ 10) were prescribed a statistically significantly higher number of unique medications than those with a low CAT score (CAT score < 10; Supplementary Table S3). All patients had ≥ 1 all-cause ambulatory visits during the baseline period, with most patients having at least one visit to the physician’s office (Supplementary Table S3). Nearly 1 in 4 patients experienced an inpatient hospitalization (22.6%), with an average length of stay for inpatient admissions of 13 days. Nearly half (45.3%) of patients had ≥ 1 ED visit, and the average number of visits among those with any ED visit was 2.1 visits per person.

### COPD-Related HCRU

Almost all patients (97.7%) had ≥ 1 COPD-related ambulatory visits during the 12-month baseline period, including 89.4% of patients requiring a physician office visit and 49.0% with at least 1 outpatient visit (Table 3). The proportion of patients with physician office visits was statistically significantly lower among patients with high disease burden compared with those with low disease burden as measured by total CAT score (88.1% vs 96.8%, *P *= 0.044). Of those patients with at least one physician office visit, patients with higher CAT scores required more visits than those with lower CAT scores [mean (SD) number of visits: 3.7 (2.4) vs 3.2 (1.8), *P *= 0.035]. No statistically significant difference was observed between the numbers of patients requiring outpatient visits.

**Table 3:** Association between COPD-cause healthcare resource utilization during the 12-month baseline period and burden of illness

	Total (*n *= 433)	Low impact (CAT 0–9) (*n *= 63)	High impact (CAT 10–40) (*n *= 370)	*p* value
Number of pharmacy fills^a^, mean (SD)	1.0 (0.0)	1.0 (0.0)	1.0 (0.0)	–
Ambulatory visits				
Proportion of patients, *n* (%)	423 (97.7)	63 (100.0)	360 (97.3)	0.370
Number, mean (SD)	5.0 (4.1)	4.7 (2.8)	5.0 (4.3)	0.502
Number among patients with ≥ 1 visit, mean (SD)	5.1 (4.1)	4.7 (2.8)	5.2 (4.3)	0.318
Office visits				
Proportion of patients, *n* (%)	387 (89.4)	61 (96.8)	326 (88.1)	0.044
Number, mean (SD)	3.3 (2.5)	3.1 (1.8)	3.3 (2.6)	0.416
Number among patients with ≥ 1 visit, mean (SD)	3.7 (2.3)	3.2 (1.8)	3.7 (2.4)	0.035
Outpatient visits				
Proportion of patients, *n* (%)	212 (49.0)	34 (54.0)	178 (48.1)	0.416
Number, mean (SD)	1.7 (3.6)	1.7 (2.5)	1.7 (3.8)	0.881
Number among patients with ≥ 1 visit, mean (SD)	3.5 (4.5)	3.1 (2.6)	3.6 (4.8)	0.396
ED visits				
Proportion of patients, *n* (%)	110 (25.4)	8 (12.7)	102 (27.6)	0.012
Number, mean (SD)	0.4 (1.0)	0.2 (0.5)	0.4 (1.0)	0.002
Number among patients with ≥ 1 visit, mean (SD)	1.6 (1.4)	1.4 (0.7)	1.6 (1.4)	0.653
IP stays (all patients)				
Proportion of patients, *n* (%)	92 (21.3)	16 (25.4)	76 (20.5)	0.406
Number, mean (SD)	0.3 (0.7)	0.4 (0.7)	0.3 (0.7)	0.428
Duration, days, mean (SD)	2.8 (9.9)	6.5 (21.1)	2.1 (6.2)	0.108
IP stays (patients with ≥ 1 admission)				
Number, mean (SD)	1.4 (0.7)	1.4 (0.7)	1.4 (0.8)	0.937
Duration, days, mean (SD)	13.1 (18.2)	25.6 (36.2)	10.4 (10.1)	0.117

*CAT* COPD Assessment Test, *COPD* chronic obstructive pulmonary disease, *ED* emergency department, *IP* inpatient, *SD* standard deviation

^a^COPD-related treatment claims

A quarter (25.4%) of patients had a COPD-related ED visit during the baseline period; the mean number of ED visits among these patients was 1.6 (Table 3). The proportion of patients with ED visits was statistically significantly higher among patients with high symptom burden (CAT score ≥ 10) than those with low symptom burden (CAT score < 10; 27.6% vs 12.7%, *P *= 0.012). Overall, 21.3% of patients were hospitalized at least once for COPD; among these patients, the mean number of hospitalizations was 1.4 with an average duration of 13 days. Differences in the numbers of inpatient visits, and the mean duration of inpatient stays, between patients with low and high CAT total scores were not statistically significant.

## Discussion

In this claims-linked survey study assessing patient-reported symptoms and burden of illness among patients with COPD treated with LAMA monotherapy, COPD had a considerable impact on patient well-being and was associated with substantial resource burden. These results are consistent with observations from previous studies which have reported that patients receiving long-acting bronchodilator monotherapy continued to experience a high symptom burden, had recent exacerbations and exhibited poor QoL, and had a higher than average rate of physician interactions [9, 24]. The majority of patients in this study experienced substantial symptom burden as measured by multiple PROs; in particular, there were high levels of dyspnea, with almost half of the patients experiencing severe dyspnea using the definitions presented in the GOLD strategic report [2]. Dyspnea poses significant problems for patients, not only in terms of day-to-day QoL but also as a marker of disease progression: for example, dyspnea is a predictor for hospitalization [25] and was found elsewhere to be more strongly correlated with 5-year survival rate than forced expiratory volume in 1 s [26].

The current analysis closely mirrored the GOLD strategy in identifying the impact of COPD in patients with a low and high level of symptoms as defined by a CAT score of < 10 or ≥ 10, respectively. In the cohort identified in this study, the vast majority of patients with COPD had a high level of symptoms. Consequently, future real world studies could plan to explore additional cut-off points in CAT score ≥ 10 to gain additional insights on the level of symptoms likely to further extenuate any increased risk of HCRU.

In this study, patients utilized a wide range of healthcare resources requiring hospitalization and ED visits, as well as a range of ambulatory visits. The majority of patients (97.7%) were seen by a healthcare professional for their COPD (office or outpatient visit) during the 12-month baseline period. A statistically significantly greater proportion of patients with lower CAT total scores (< 10) had a physician office visit compared with patients with higher CAT scores (≥ 10). Conversely, patients with higher CAT scores had statistically significantly more ED visits, suggesting greater disease severity or suboptimal management, consistent with the greater symptom burden experienced by this group.

In view of the considerable symptom burden and resource use evident among patients treated with LAMA monotherapy in this study, it is possible that many of these patients could benefit from escalation of therapy. In accordance with the current GOLD recommendations, clinicians should consider the use of an additional bronchodilator such as LAMA/LABA combination therapy when a monotherapy bronchodilator does not provide adequate symptom control [2]. Multiple clinical trials and network meta-analyses have reported improved lung function and QoL outcomes with the use of LAMA/LABA combination therapy compared with long-acting bronchodilator monotherapy [27–34].

It should be noted that CAT, mMRC, and EXACT assess different aspects of COPD burden of illness, and it remains unclear which of these tools or combination of tools should be prioritized in assessment of patient symptoms. The current study was limited to patients receiving LAMA monotherapy and excluded all patients prescribed ICS or dual bronchodilators. The reasons why patients with poor disease control on monotherapy did not escalate to combination therapies were not evaluated. One possible reason might be an underestimation of the symptom burden by the physician, as identified by Mapel et al. [35] or poor communication of the symptom burden between patients and physicians.

We note that the study population is slightly older, has a higher proportion of female patients, and a lower percentage of smokers than are usually seen in COPD clinical trials [36]. In addition, a high proportion of patients have comorbidities, in particular those related to ASCVD, [36] therefore it is possible that dyspnea observed in these patients may not be due only to COPD.

Limitations of this study included those typically associated with claims-linked survey studies. Because claims data are collected for payment rather than research, this data source is associated with certain limitations: the presence of a claim for a filled prescription does not indicate that the medication was consumed or taken as prescribed, and medications filled over-the-counter or provided as samples by a physician were not captured. Additionally, the presence of a diagnosis code on a medical claim does not constitute conclusive evidence of the disease. Patients with a diagnosis of asthma were not excluded from the study; therefore, as no spirometry data were available to confirm the COPD diagnosis, it is possible that the diagnosis code may have been included as a rule-out criterion or incorrectly coded. To help address these limitations, multiple pharmacy claims and diagnosis codes were required for sample inclusion; the requirement for patients to have claims-based and self-reported treatment and multiple diagnosis codes for COPD at least 30 days apart ensured that a prevalent population of patients with COPD was included in the analysis. Patients were also required to report a healthcare provider COPD diagnosis and COPD treatment. The study is subject to limitations of survey data, including sampling error, coverage error, and measurement error. Finally, the study population comprised patients with commercial health plan coverage and MA enrollees, and therefore the results may not be generalizable to uninsured populations or more broadly to populations outside the USA.

## Conclusions

While LAMA monotherapy has demonstrated efficacy and has been shown to reduce dyspnea, exacerbations, and hospitalizations in patients with COPD, [37] the results of this study demonstrate that a large proportion of patients receiving LAMA monotherapy still remain symptomatic. Escalation of therapy to a dual LAMA/LABA combination may be indicated to reduce patient burden of illness and improve patient QoL, which may in turn reduce HCRU. Better utilization of PROs to understand symptom severity and disease burden in COPD may lead to improved treatment strategies and in turn to amelioration of disease burden and reduced HCRU. Physicians should therefore consider including questions or tools to measure symptom burden, such as the CAT or mMRC, as part of routine care for patients with COPD.

## Electronic Supplementary Material

Below is the link to the electronic supplementary material.

Supplementary material 1 (DOCX 24 kb)
