# Supplementary material for: Patient-Reported Burden of Illness in a Prevalent COPD Population Treated with Long-Acting Muscarinic Antagonist Monotherapy: A Claims-Linked Patient Survey Study
Source: Pulm Ther. 2019 Apr 1;5(1):69–80. doi: 10.1007/s41030-019-0091-0 (PMC6967076; doi:10.1007/s41030-019-0091-0)
Supplement: Supplementary file 1 — Supplementary material 1 (DOCX 24 kb) [file 41030_2019_91_MOESM1_ESM.docx]

**SUPPLEMENTARY MATERIALS**

**Supplementary Table S1** ICD-10-CM diagnosis codes and medications

| **ICD-10-CM diagnosis codes for COPD** | | |
| --- | --- | --- |
| **Code** | **Type** | **Description** |
| J40 | ICD-10 Dx | Bronchitis, not specified as acute or chronic |
| J410 | ICD-10 Dx | Simple chronic bronchitis |
| J411 | ICD-10 Dx | Mucopurulent chronic bronchitis |
| J418 | ICD-10 Dx | Mixed simple and mucopurulent chronic bronchitis |
| J42 | ICD-10 Dx | Unspecified chronic bronchitis |
| J430 | ICD-10 Dx | Unilateral pulmonary emphysema  (MacLeod’s syndrome) |
| J431 | ICD-10 Dx | Panlobular emphysema |
| J432 | ICD-10 Dx | Centrilobular emphysema |
| J438 | ICD-10 Dx | Other emphysema |
| J439 | ICD-10 Dx | Emphysema, unspecified |
| J440 | ICD-10 Dx | Chronic obstructive pulmonary disease |
| J441 | ICD-10 Dx | Chronic obstructive pulmonary disease with (acute) exacerbation |
| J449 | ICD-10 Dx | Chronic obstructive pulmonary disease, unspecified |
| **COPD medications** | | |
| **Subclass** | **Medication** | |
| Anticholinergic/  SABA combination | Ipratropium/albuterol | |
| Anticholinergics  (LAMA  monotherapy) | Aclidinium  Tiotropium  Umeclidinium | |
| ICS | Beclomethasone  Budesonide  Ciclesonide  Dexamethasone  Flunisolide  Fluticasone  Mometasone  Triamcinolone | |
| LABA | Arformoterol  Formoterol  Olodaterol  Salmeterol  Indacaterol | |
| LAMA/LABA combination | Umeclidinium/vilanterol  Tiotropium/olodaterol  Glycopyrrolate/indacaterol | |
| ICS/LABA combination | Fluticasone/salmeterol  Budesonide/formoterol  Fluticasone/vilanterol  Mometasone/formoterol | |

*ICD-10-CM* International Classification of Disease, 10^th^ Revision-Clinical Modification; *ICS* inhaled corticosteroid; *LABA* long-acting β_2_-agonist; *LAMA* long-acting muscarinic antagonist; *MMMC* mainstream Medicaid managed care; *SABA* short-acting β_2_-agonist

**Supplementary Table S2** Baseline comparison of respondents and non-respondents claim-based characteristics

|  | **Total**  **(N=2,275)** | **Respondents (n=654)** | **Non-respondents**  **(n=1,621)** | ***P*-value** |
| --- | --- | --- | --- | --- |
| **Age (years), mean (SD)** | 72.1 (10.0) | 71.5 (9.0) | 72.3 (10.4) | 0.071 |
| **Female, n (%)** | 1,343 (59.0) | 386 (59.0) | 957 (59.0) | 0.994 |
| **Insurance type, n (%)** |  |  |  |  |
| Commercial | 295 (13.0) | 70 (10.7) | 225 (13.9) | 0.041 |
| Medicare Advantage | 1,980 (87.0) | 584 (89.3) | 1,396 (86.1) | 0.041 |
| **Geographic region, n (%)**  Northeast | 476 (20.9) | 118 (18.0) | 358 (22.1) | 0.032 |
| Midwest | 607 (26.7) | 178 (27.2) | 429 (26.5) | 0.714 |
| South | 955 (42.0) | 292 (44.7) | 663 (40.9) | 0.101 |
| West | 237 (10.4) | 66 (10.1) | 171 (10.6) | 0.747 |

*SD* standard deviation

**Supplementary Table S3** Association between all-cause healthcare resource utilization during the 12‑month baseline period and burden of illness

|  | **Total**  **(N=433)** | **Low Impact**  **(CAT 0–9) (n=63)** | **High Impact (CAT 10–40)**  **(n=370)** | ***P*-value** |
| --- | --- | --- | --- | --- |
| **Count of unique medications, mean (SD)** | 12.8 (6.7) | 10.3 (4.5) | 13.2 (6.9) | <0.001 |
| **Ambulatory visits** |  |  |  |  |
| proportion of patients, n (%) | 433 (100.0) | 63 (100.0) | 370 (100.0) | - |
| number, mean (SD) | 23.0 (16.7) | 23.3 (18.8) | 23.0 (16.3) | 0.884 |
| number among patients with  ≥1 visit, mean (SD) | 23.0 (16.7) | 23.3 (18.8) | 23.0 (16.3) | 0.884 |
| **Office visits** |  |  |  |  |
| proportion of patients, n (%) | 425 (98.2) | 62 (98.4) | 363 (98.1) | 1.000 |
| number, mean (SD) | 14.9 (12.4) | 16.1 (16.8) | 14.7 (11.5) | 0.530 |
| number among patients with  ≥1 visit, mean (SD) | 15.2 (12.3) | 16.4 (16.8) | 15.0 (11.4) | 0.541 |
| **Outpatient visits** |  |  |  |  |
| proportion of patients, n (%) | 341 (78.8) | 52 (82.5) | 289 (78.1) | 0.507 |
| number, mean (SD) | 8.1 (10.6) | 7.2 (6.6) | 8.3 (11.2) | 0.294 |
| number among patients with  ≥1 visit, mean (SD) | 10.3 (11.0) | 8.8 (6.3) | 10.6 (11.6) | 0.094 |
| **ED visits** |  |  |  |  |
| proportion of patients, n (%) | 196 (45.3) | 25 (39.7) | 171 (46.2) | 0.412 |
| number, mean (SD) | 1.0 (1.8) | 0.7 (1.0) | 1.0 (1.8) | 0.054 |
| number among patients with  ≥1 visit, mean (SD) | 2.1 (2.1) | 1.8 (0.9) | 2.2 (2.2) | 0.086 |
| **IP stays (all patients)** |  |  |  |  |
| proportion of patients, n (%) | 98 (22.6) | 16 (25.4) | 82 (22.2) | 0.625 |
| number, mean (SD) | 0.3 (0.7) | 0.4 (0.8) | 0.3 (0.7) | 0.382 |
| duration, days, mean (SD) | 2.9 (10.1) | 6.8 (21.4) | 2.3 (6.3) | 0.101 |
| **IP stays (patients with ≥1 admission)^a^** |  |  |  |  |
| number, mean (SD) | 1.4 (0.8) | 1.6 (0.7) | 1.4 (0.8) | 0.472 |
| duration, days, mean (SD) | 13.0 (18.0) | 26.8 (36.4) | 10.3 (10.0) | 0.092 |

^a^A subanalysis was carried out on 98 patients who required ≥1 inpatient stay during the 12-month baseline period.

*CAT* COPD Assessment Test; *COPD* chronic obstructive pulmonary disease; *ED* emergency department; *IP* inpatient; *SD* standard deviation
